# The Cytochrome P450 gene *CYP6P12* confers pyrethroid resistance in *kdr*-free Malaysian populations of the dengue vector *Aedes albopictus*

**DOI:** 10.1038/srep24707

**Published:** 2016-04-20

**Authors:** Intan H. Ishak, Jacob M. Riveron, Sulaiman S. Ibrahim, Rob Stott, Joshua Longbottom, Helen Irving, Charles S. Wondji

**Affiliations:** 1 Department of Vector Biology, Liverpool School of Tropical Medicine, Pembroke Place, Liverpool L3 5QA, United Kingdom; 2 School of Biological Sciences, Universiti Sains Malaysia, 11800 Penang, Malaysia

## Abstract

Control of *Aedes albopictus*, major dengue and chikungunya vector, is threatened by growing cases of insecticide resistance. The mechanisms driving this resistance remain poorly characterised. This study investigated the molecular basis of insecticide resistance in Malaysian populations of *Ae. albopictus*. Microarray-based transcription profiling revealed that metabolic resistance (cytochrome P450 up-regulation) and possibly a reduced penetration mechanism (consistent over-expression of cuticular protein genes) were associated with pyrethroid resistance. *CYP6P12* over-expression was strongly associated with pyrethroid resistance whereas *CYP6N3* was rather consistently over-expressed across carbamate and DDT resistant populations. Other detoxification genes also up-regulated in permethrin resistant mosquitoes included a glucuronosyltransferase (AAEL014279-RA) and the glutathione-S transferases GSTS1 and GSTT3. Functional analyses further supported that *CYP6P12* contributes to pyrethroid resistance in *Ae. albopictus* as transgenic expression of *CYP6P12* in *Drosophila* was sufficient to confer pyrethroid resistance in these flies. Furthermore, molecular docking simulations predicted *CYP6P12* possessing enzymatic activity towards pyrethroids. Patterns of polymorphism suggested early sign of selection acting on *CYP6P12* but not on *CYP6N3*. The major role played by P450 in the absence of *kdr* mutations suggests that addition of the synergist PBO to pyrethroids could improve the efficacy of this insecticide class and overcome resistance in field populations of *Ae. albopictus.*

The mosquito *Aedes albopictus* Skuse is an important vector of dengue and chikungunya throughout the tropical and subtropical world. Although it has lower oral receptivity for dengue virus compared with *Ae. aegypti*^1^, it plays an important role in maintaining the dengue virus by transmitting it through mating and transovarial transmission^2^,^3^. In the absence of *Ae. aegypti*, dengue outbreaks could be caused by *Ae. albopictus*^4^ as observed in China where this species was the cause of dengue outbreaks in 2004^5^. *Ae. albopictus* is also playing a major role in chikungunya transmission in many countries such as in La Réunion island^6^.

Control of *Ae. albopictus* relies on insecticide-based interventions such as Ultra Low Volume (ULV) space sprays, fogging and thermal spraying. Although there are less reports of insecticide resistance in *Ae. albopictus* than in *Ae. aegypti,* cases of resistance are increasingly reported for this species. Resistance towards DDT has been recorded in adult *Ae. albopictus* populations from Cameroon^7^ and Sri Lanka^8^,^9^,^10^. In Sri Lanka, adult *Ae. albopictus* populations were also moderately resistant towards 0.8% malathion^8^. However, this species have been shown to be susceptible to carbamates (propoxur) and pyrethroids either permethrin or deltamethrin in India, Thailand, Greece and Italy^10^. In Malaysia, after previous reports of susceptibility to pyrethroids^11^,^12^ recent studies have detected the presence of resistance in *Ae. albopictus* against permethrin and deltamethrin. First, pyrethroid resistance was reported in larvae in Kuala Lumpur^13^ and second, a recent assessment of the susceptibility profile of this species across Malaysia showed that although it was less resistant than *Ae. aegypti*, it nevertheless was resistant to several insecticides such as carbamates, organophosphates and organochlorines (DDT, dieldrin). Additionally, a population from Kuala Lumpur was resistant to pyrethroids^14^. No *kdr* was detected in these populations suggesting that metabolic resistance could be the main resistance mechanism^14^. However, contrary to *Ae. aegypti* for which significant progress has been made^15^,^16^,^17^,^18^, little is known about the molecular basis of insecticide resistance in *Ae. albopictus* partly because progress has been slow in developing genetics and genomics tools for this vector compared to *Ae. aegypti*. Recent genomics tools have nevertheless been designed in *Ae. albopictus* such as a de novo transcriptome^19^ and the first draft genome^20^ opening the possibility for more in-depth and genome-wide analysis of the molecular basis of various phenotypes such as insecticide resistance in this important arbovirus vector.

This study has investigated the molecular basis of pyrethroid resistance, in field populations of *Ae. albopictus* in Malaysia. Using a microarray-based genome-wide transcription profiling and functional analyses, we demonstrated that the up-regulation of the cytochrome P450s, particularly *CYP6P12,* is conferring pyrethroid resistance in Malaysian *Ae. albopictus*. Furthermore, this study also revealed that a reduced penetration mechanism may be associated with pyrethroid resistance with consistent over-expression of cuticular protein genes.

## Results

### Susceptibility profiles of *Ae. albopictus* populations analysed

**Figure 1:**
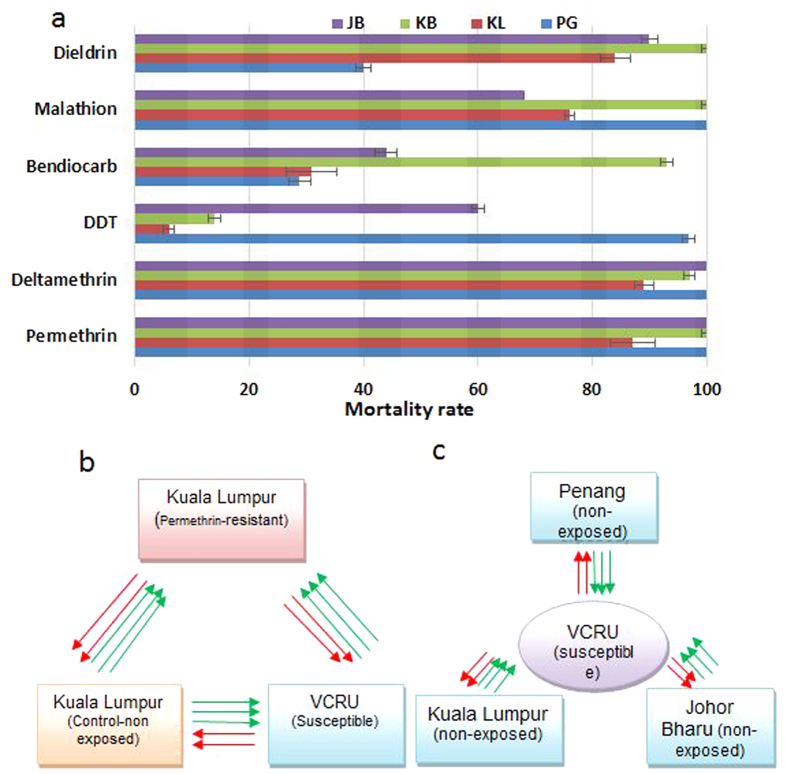
Insecticide resistance profile and experimental design of transcriptomic analyses. (**a**) Resistance profiles to different insecticide classes in female *Ae. albopictus* mosquitoes across Malaysia including KL (Kuala Lumpur), PG (Penang), JB (Johor Bharu) and KB (Kota Bharu). (**b**) Schematic representation of the experimental design of the microarray studies for the permethrin resistance profiling *whereas* (**c**) is for the comparison between non-exposed (control) vs susceptible samples across Malaysia. Green arrows refer to Cy3 dye, and red arrows refer to Cy5 dye.

The *Ae. albopictus* populations from Malaysia used in this study were fully susceptible to pyrethroids (both type I and II) except in Kuala Lumpur where a resistance was observed to permethrin and to deltamethrin (87% and 89% mortality respectively) (Fig. 1a). For DDT, a mixed resistance pattern was observed with Kuala Lumpur and Kota Bharu exhibiting high resistance levels (6 and 14% mortality rate respectively) while the Penang population was nearly fully susceptible (96.8% mortality)(Fig. 1a). In contrast, most populations were resistant to bendiocarb except for Kota Bharu (93% mortality). Overall, the KL population was the most resistant as it was also resistant to all insecticide classes including to the organophosphate malathion and to the organochlorine dieldrin (Fig. 1a).

### Genome-wide microarray-based transcriptional profiling

The transcriptional profiles of resistant *Ae. albopictus* populations in Malaysia were analyzed with two aims; i) first to detect the genes directly associated with permethrin resistance through a comparison of the sets of genes differentially expressed between the permethrin R-C, R-S and C-S hybridizations from KL (Fig. 1b); ii) Second, to detect the genes associated with resistance to other insecticides (such as bendiocarb, DDT and malathion) across Malaysia through a comparison of the C-S hybridizations between KL, PG and JG (Fig. 1c). The complete sets of differentially expressed probes from all comparisons in this study are presented in dataset 1.

### 1-Pyrethroid resistance mechanisms

**Figure 2:**
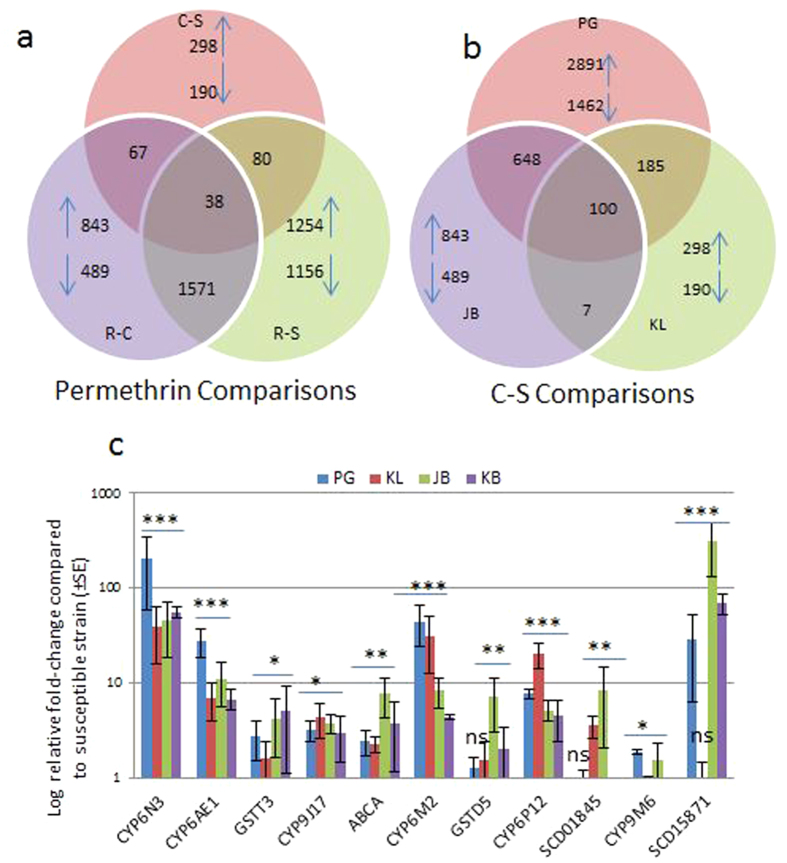
Transcription profiling of *Ae. albopictus* in Malaysia: (**a**) Summary of probes differentially expressed for permethrin comparisons for the Kuala Lumpur (KL) population, whereas (**b**) is for C-S comparisons from KL (Kuala Lumpur), PG (Penang) and JB (Johor Bharu). The Venn diagrams show the number of probes (or ESTs) significantly (P value < 0.01) up- or down-regulated (>2 fold change) in each comparison as well as the commonly expressed probes. Upward arrows indicate up-regulated probes while downward represent down-regulated. (**c**) Differential expression by qRT-PCR of 11 genes up-regulated in microarray in the four locations. Statistical significance is indicated by ***for P < 0.001; **for P < 0.01: *for P < 0.05 and ns is for non-significant of fold-change in comparison to the susceptible strain VCRU.

Due to the major role of pyrethroid in ongoing vector control interventions against dengue vectors in Malaysia, emphasis was put in investigating the underlying metabolic resistance mechanisms against permethrin. The KL sample was chosen for this study as it was the only location where *Ae. albopictus* was resistant to permethrin^14^. In addition, the relatively lower level of permethrin resistance in KL also makes it more relevant to compare resistant mosquitoes to non-exposed control sample from KL. The large phenotypic difference between the permethrin resistant mosquitoes (100% resistant, since all survivors are considered resistant) and the control non-exposed (only 13% resistant since there was a mortality of 87% after exposure to permethrin) present a higher contrast likely to facilitate the detection of permethrin resistance genes by microarray hybridisation between resistant (R) and Control (C) (R-C) as there is no confounding factor caused by the difference in genetic background. Furthermore, the permethrin resistant mosquitoes (R) were also compared to the lab permethrin susceptible (S) VCRU strain (R-S) to further validate the consistency of the expression pattern of candidate resistance genes. The possible induction of some genes in the R-C and R-S comparisons because of the exposure to insecticide was corrected by also performing a comparison of the control non exposed mosquitoes (C) to the susceptible ones (S) (C-S). Overall, the expression profiles from the three R-C, R-S and C-S hybridisations were used to generate the list of candidate resistance genes as presented in Table S1. The number of differentially expressed probes was determined with a cut-off of >2-fold at P < 0.01 for each comparison as indicated in Fig. 2a.

#### Genes up-regulated in permethrin resistant mosquitoes (R-C)

More attention was paid to the R-C comparison since it directly reflects a difference in permethrin resistance phenotype without difference in genetic background. The most up-regulated transcript, Aalb_oocyte_GH79BIP02H77ZJ (FC 77.3), belongs to a cuticular protein gene from the CPLCG family (Table S1) with closest BLAST hit to the *An. gambiae* AGAP008449 (CPLCG5) (98%) and AGAP008446 (CPLCG3) (96%) previously suggested to be associated to a reduced penetration resistance in field population of this malaria vector in Africa^21^. This transcript was also up-regulated in the R-S comparison. The possible involvement of a reduced penetration mechanism in the permethrin resistance of this KL population was further supported by the presence of several other probes belonging to cuticle protein genes with high FC (Table S1) with four of the five top up-regulated resistance-related genes belonging to cuticular protein genes. Furthermore, the consistency of the up-regulation of cuticular protein genes was highlighted by the fact that their up-regulation was observed for probes designed from both *Ae. albopictus* ESTs and *Ae. aegypti* transcripts.

Probes from the cytochrome P450s were the most predominant among the detoxification genes (Table S1). The P450 transcript Aalb_oocyte_ GH79BIP02GBWB9, with the closest hit to *CYP6P12* in *Ae. aegypti* (89%) or 74% similarity to *CYP6P4* in *An. gambiae,* was the most over-expressed cytochrome P450 (FC 31.8). Furthermore, three probes of another *Ae. albopictus* transcript HQ621849.1 that also has the closest hit to *CYP6P12* in *Ae. aegypti* was consistently over-expressed with FC values of 11.36, 11.16 and 8.76. These probes were also up-regulated in R-S. Additionally, several other P450 transcripts such as JF317339.1 also had the closest hit corresponding to the *CYP6P4* gene in *An. gambiae* (Table S1). Other cytochrome P450s that were up-regulated were genes with the closest hit to *Ae. aegypti CYP6ZB1* (Aalb_oocyte_GIK0NFC01EFN86 and Aalb_oocyte_rep_c13705), *CYP6AG6* (Aalb_oocyte_rep_c3445 and Aalb_oocyte_rep_c28874), *CYP6A1*, *CYP6Z8* and *CYP9J6* (Table S1).

A set of five transcripts belonging to GSTs was also up-regulated in the R-C comparison with the *GSTS1* gene (AAEL011741-RB) being the most over-expressed FC 20.2) follow by *GSTT3* (Aalb_oocyte_rep_c11155) (FC 16.5) and *GSTT4* (FC 5.1). However, the most over-expressed detoxification gene in R-C was the phase II enzyme *UDP glucoronosyltransferase* (AAEL014279-RA), with a high FC of 60.7 despite the corresponding probes being designed from the sequence of an *Ae. aegypti* transcript. Other detoxification or resistance related genes are reported in Table S1 including the *aldehyde oxidase* (AAEL0010384), *glycogenin* (AAEL014863), *short-chain dehydrogenase* (AAEL008227-RA), or two alpha-esterase genes Aalb_oocyte_rep_c21955 (with 82% similarity to AAEL013543) and AAEL015264-RA.

#### Commonly up-regulated genes between comparisons

Only a limited number of genes were commonly up-regulated in the three comparisons. A single P450, *CYP6AG6,* was common to R-C, R-S and C-S comparisons (Table S1). Interestingly the antimicrobial peptide Holotricin, which is the top up-regulated gene in the C-S comparison, is consistently up-regulated in the three comparisons (Table S1). Noticeably, 3 probes for the P450 gene *CYP6N3* were consistently, commonly over-expressed in C-S and also R-S comparisons but not in the R-C, suggesting that this gene could be involved in resistance to other insecticides but is less likely to play any role in pyrethroid resistance.

#### Genes commonly down-regulated between comparisons

Out of the top genes commonly down-regulated between the three comparisons, *serine threonine-protein kinase* is the one the most down-regulated. *4-nitrophenylphosphatase* seems to be consistently down-regulated with the probes representing this gene to be among the top 4 probes (Table S2).

### 2-Detection of insecticide resistance genes across Malaysia using C-S

In order to detect the main genes associated with resistance to other insecticide classes than pyrethroids across Malaysia, C-S comparisons were analysed from PG, KL and JB samples. The number of probes differentially expressed in each location are indicated in Fig. 2b.

#### Genes commonly up-regulated in the three locations

The most over-expressed gene in the three locations was Holotricin, an antimicrobial peptide (Table S3). The detoxification genes included only three cytochrome P450s (Table 1 and S3) among which transcripts corresponding to *CYP6N3* (HQ621851), *CYP9AE1* (Aalb_oocyte_rep_c24780) and *CYP6AG6* (AAEL006992-RA). Since bendiocarb resistance is the common resistance observed in these 3 populations, one can suggest that the genes could be associated to this resistance. Other probes belong to other gene families such as redox families, protein synthesis, ion transport or immune responses.

#### Genes commonly up-regulated in KL and PG but not in JB

The set of detoxification genes commonly over-expressed in KL and PG includes only 3 cytochrome P450s. The most over-expressed probes of these P450s belong to *CYP6N3* with three probes with a higher expression consistently observed in PG than in KL. The two other P450s are *CYP6AG6* (Aalb_oocyte_rep_c28874 transcript) and *CYP6AL3* (AAEL009656-RA). Other over-expressed genes from this list belong to alcohol dehydrogenase, hydrolase, juvenile hormone, and others (Table 1).

#### Genes commonly up-regulated in PG and JB but not in KL

More genes were commonly over-expressed in PG and JB than between PG and KL. From the 34 detoxification probes over-expressed, more than half belong to P450 genes (20), others to GSTs (5), ABC transporters (4), carboxylesterase (1) and proteases. Probes from over-expressed CYP6 P450s included 4 probes from *CYP6N3*, 5 probes from P450 transcripts with closest hit to *An. gambiae CYP6P4*, 5 probes from the *Ae. aegypti CYP6P12* and probes from the *Ae. aegypti* genes *CYP6Z6*, *CYP6Z8* and *CYP6ZB1*. The two CYP9 genes over-expressed were *CYP9AE1* and *CYP9M6* (as annotated in *Ae. aegypti*) (Table 1). The 5 over-expressed GST probes belong to three genes with the closest hit to the *Ae. aegypti GSTT3* (1 probe), *GSTD5* (2 probes) and *GSTE3* (2 probes). A consistent higher over expression of all the GSTs was observed in JB than PG. The top up-regulated GST in JB was *GSTD5* with FC value of 13.73 but an FC of 4.36 in PG (Table 1). The unique carboxylesterase gene (Aalb_oocyte_GIK0NFC01CWBYU) showed a similarly low expression in both PG (FC 2.44) and JB (FC 2.34) (Table 1). The higher number of genes commonly over-expressed between JB and PG could reflect the presence of similar resistance mechanisms between the two location as they present a similar resistance profile than with KL^14^.

No significantly over-expressed probes were found to be commonly over-expressed only between KL and JB, whereas the list of probes over-expressed in a single location consists of a mixture of cytochrome P450s, GSTs, ABC transporters, cuticle proteins and also several proteases (Table S4).

#### Genes commonly down-regulated in the three locations

Out of the top genes commonly down-regulated in PG, JB and KL, *vitellogenin* and *vitelline protein* seems to be consistently down-regulated. Probes with unknown function were amongst the top commonly down-regulated (Table S5).

### Validation of candidate genes through qRT-PCR

The expression pattern of eleven genes among the most over-expressed detoxification genes and belonging to various detoxification gene families from microarray was further validated by qRT-PCR including six cytochrome P450s (*CYP6N3*, *CYP6AE1*, *CYP6P12*, *CYP9M6*, *CYP9J17*, the ortholog of the *An. gambiae CYP6M2*), two glutathione-S transferases (*GSTT3*, *GSTD1*), one ABC transporter (*ABC transporter A*) and two short-chain dehydrogenases SCD15871 (Aalb_oocyte_rep_c15871) and SCD01845 (AAEL001845-RA) (Fig. 2c). Samples from Kota Bharu (KB) were also tested in the qRT-PCR. A significant over-expression in all four populations was confirmed for 10 genes except for *CYP9M6* when their relative expression was compared between the four populations and susceptible VCRU strain (Fig. 2c). Overall, a high level of correlation was observed between both results with a R^2^ of 0.85 observed in PG (Figure S1). The cytochrome P450 *CYP6N3* was the most consistently, top, over-expressed gene across the four locations with FC value of 201.4 in PG, 39.8 in KL, 45.3 in JB and 55.1 in KB, corresponding to similar patterns obtained from the microarray.

### Transgenic expression of *CYP6P12* in *Drosophila* flies

**Figure 3:**
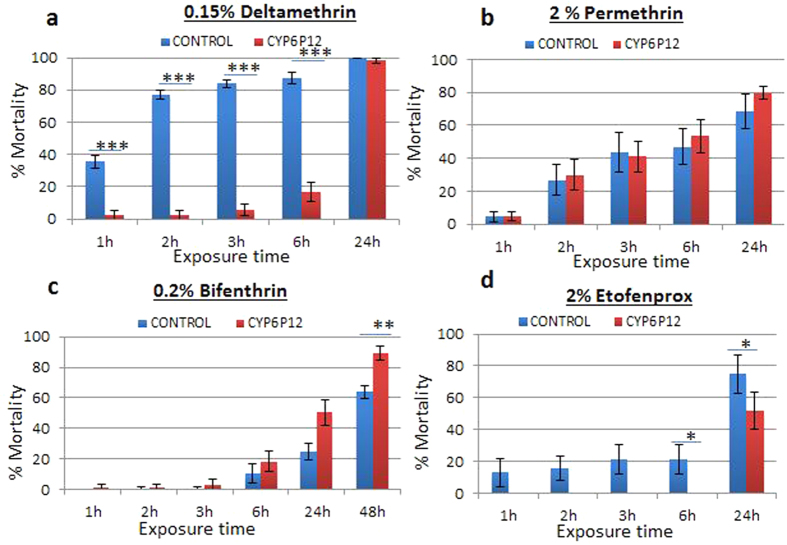
Knockdown and mortality rate after bioassay tests with transgenic strains for CYP6P12 at different time-points. (**a**) is the result for test with deltamethrin on the transgenic Act5C-CYP6P12 strain and the control strain (the progeny from the cross between the UAS-CYP6P12 females and w^1118^ males (which do not over-express the P450 transgene). (**b**) is the result for permethrin on transgenic Act5C-CYP6P12; (**c**) is the result for bifenthrin on transgenic Act5C-CYP6P12 and (**d**) is for etofenprox on transgenic Act5C-CYP6P12.

To confirm that the over-transcription of *CYP6P12* alone can confer pyrethroid resistance, transgenic *D. melanogaster* expressing a *CYP6P12* allele from KL (Figure S2) under the control of the ubiquitous Act5C-GAL4 driver were successfully generated. Contact bioassays performed with 0.15% deltamethrin (Type II pyrethroid) revealed that transgenic flies over-expressing *CYP6P12* were resistant to deltamethrin with significantly reduced mortality/knockdown rates observed at four different exposure times compared with the control group not expressing *CYP6P12.* These exposure times are 1 h (2.5 vs. 35.5%, P = 0.0004), 2 h (2.5 vs. 77%, P < 0.0001), 3 h (5.8 vs. 87.5%, P < 0.0001) and 6 h (16.8 vs. 87.5%, P < 0.0001) (Fig. 3a; Table S6). This results demonstrate that over-transcription of *CYP6P12* alone is sufficient to confer resistance to deltamethrin.

For type I pyrethroids, a different pattern was observed as transgenic flies rather exhibited a similar or significantly higher knockdown/mortality rates than control for both permethrin and bifenthrin. Indeed, although not statistically significant (P = 0.31), bioassays with 2% permethrin revealed a higher mortality of 79.7% in transgenic Act5C-CYP6P12 flies than in control group not expressing *CYP6P12* (68.4%) after 24 h (Fig. 3b). This phenomenon was even more pronounced for bifenthrin for which the transgenic flies exhibited after 48 h exposure to 0.2% bifenthrin papers, a significantly higher mortality rate (P = 0.0026) was observed in transgenic flies expressing CYP6P12 (89.2% mortality) than in control flies with no expression (61.1%) (Fig. 3c; Table S6). These results could suggest that over-expression of *CYP6P12* rather increased toxicity to type I pyrethroids suggesting that *CYP6P12* is probably metabolizing these insecticides but into more toxic metabolites.

Further bioassays performed with the ether pyrethroid, etofenprox, also showed that transgenic flies expressing CYP6P12 are resistant to this pseudo-pyrethroid, with significantly lower mortality in transgenic flies than control after 3 and 6 h exposure to 2% etofenprox (0% vs. 21.3%; P = 0.049) (Fig. 3d). Pyrethroid bioassays performed with male flies presented similar results to that of females (Table S6).

Attempt was made to assess whether *CYP6P12* confers cross-resistance to the carbamate bendiocarb exposing transgenic Act5C-CYP6P12 to 0.01% bendiocarb paper. Transgenic female flies expressing *CYP6P12* showed no significant difference in rates of mortality at any time point when compared with the control female group (88.2% and 98.3% mortality after 24 hours respectively, P = 0.23; Figure S3a; Table S6). Male transgenic *Drosophila* expressing *CYP6P12* did however show a significant difference in rate of mortality after 2 hours of exposure when compared with the control male group (41.6% vs 100% mortality respectively; P = 0.0096; Figure S3b). However, this was the only time point showing significant difference.

### Polymorphism analysis of *CYP6P12* and *CYP6N3*

**Figure 4:**
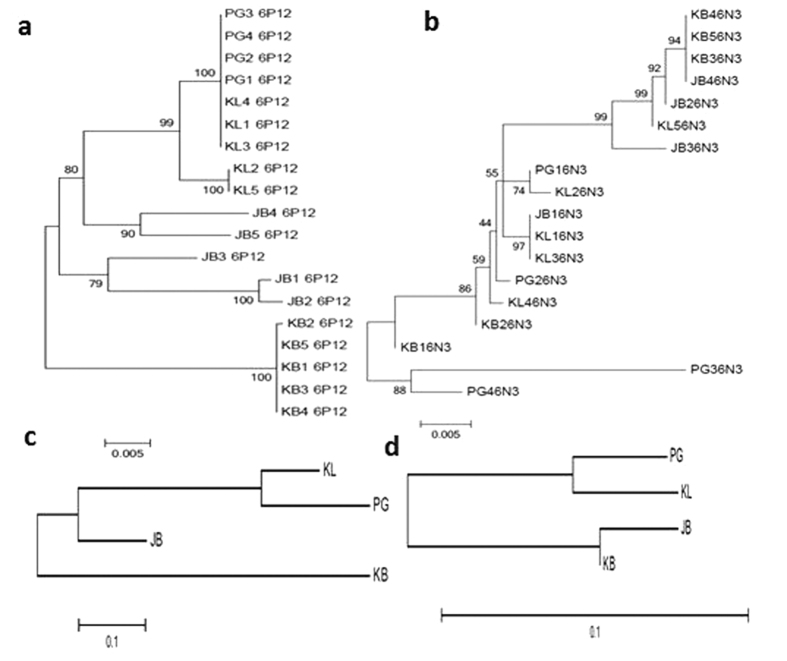
Genetic diversity patterns of *CYP6P12* and *CYP6N3* across Malaysia from KL (Kuala Lumpur), PG (Penang), JB (Johor Bharu) and KB (Kota Bharu): (**a**) Maximum likelihood tree of *CYP6P12,* while (**b**) is for *CYP6N3;* (**c**) Genetic distance between populations across Malaysia based on *CYP6P12 Nst* estimates while (**d**) is for C*YP6N3*.

Analysis of the polymorphism patterns of the full-length cDNA sequences of *CYP6P12* (1527 bp) *and CYP6N3* (1500 bp) in the four populations in Malaysia revealed that both genes are highly polymorphic across Malaysia with a high number of substitution sites (129 and 88 respectively for *CYP6P12* and *CYP6N3*) (Table S7). However, if the polymorphism level is similar between locations for *CYP6N3*, three of the four locations (KL, PG and KB) present a reduced diversity for the *CYP6P12* genes as shown by lower substitution sites (0 to 15 compared to 85 in JB), lower number of haplotypes (1 to 2 compared to 5 in JB) possibly suggesting that a selection could be acting on this gene as a consequence of ongoing resistance. This is further supported for the KL sample for which a significant estimate of the neutrality test D* (Fu and Li) was observed. This could be associated with the pyrethroid resistance observed in KL. However, further analysis with more sequences is needed to establish the presence of such signature of selective sweep. A maximum likelihood phylogenetic tree of both genes revealed that *CYP6P12* haplotypes cluster according to their location of origin with those from KL clustering with PG haplotypes but differently from JB and KB (Fig. 4a). In contrast, CYP6N3 appear to cluster randomly in accordance to the consistent high polymorphism in all locations (Fig. 4b). Overall, the level of genetic differentiation between locations appears similar for *CYP6P12* and *CYP6N3* as the *Nst* genetic distance tree shows that PG and KL cluster together for both genes (Fig. 4c,d).

### *In silico* modeling of *CYP6P12* and docking

**Figure 5:**
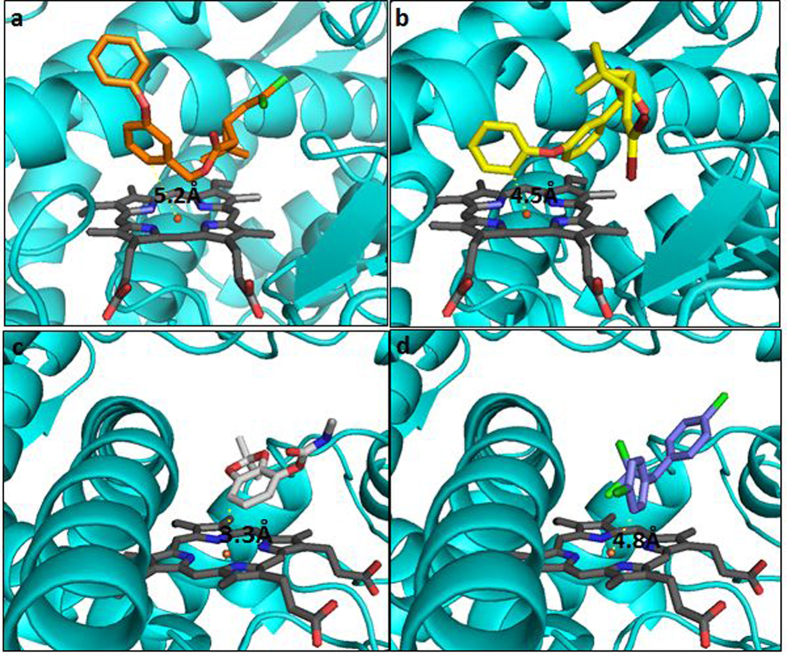
Binding conformation of (**a**) permethrin, (**b**) deltamethrin, (**c**) bendiocarb, and (**d**) DDT in the active site of CYP6P12. CYP6P12 is presented in helices and cyan in colour. Heme is in stick format and grey. Distances between insecticides and heme iron are annotated.

Docking parameters for all insecticide ligands screened are provided in Table S8. Overall, CYP6P12 produced good binding score for all pyrethroids screened, as well as DDT and bendiocarb. However, analysis of the binding conformation revealed that though pyrethroids bind productively in the active site of the enzyme, the sites of hydroxylation were not the 4′ spot established as the most preferred by the insect P450s. For example, permethrin binds with the 6 position of the benzyl ring oriented above the heme and within 5.2 Å from the heme iron, while deltamethrin binds with the 2′ spot of the phenoxy ring oriented for hydroxylation at a distance of 4.5 Å (Fig. 5a,b respectively). The 4′ phenoxy ring has been established as the most preferred site of hydroxylation of pyrethroids by insect P450^22^,^23^.

Bendiocarb binds with the aromatic group above the heme at a distance of 3.3 Å from the heme iron. In this posture aromatic ring hydroxylation was predicted (Fig. 5c). DDT productively binds with the trichloromethyl group above the heme and at a distance of 4.8 Å from the heme iron (Fig. 5d). In this posture hydroxylation is possible but the chlorine atoms projecting above the heme are predicted to result in steric clashes. DDT, however exhibited higher score than bendiocarb.

## Discussion

This study has provided a broad insight into the molecular mechanisms driving resistance to insecticides, notably pyrethroid resistance in the dengue vector *Ae. albopictus* in Malaysia, revealing that metabolic resistance and possibly a reduced penetration mechanisms are key mechanisms.

### 1-Pyrethroid resistance is likely under the control of cytochrome P450s and a reduced cuticle penetration mechanism

The transcription analysis revealed that permethrin resistance in *Ae. albopictus* in KL was under the control of a metabolic resistance and likely a reduced penetration through the over-expression of cytochrome P450s and cuticular protein genes respectively. Because of a greater phenotypic contrast between the permethrin resistant (R) and the unexposed mosquitoes (C) due the moderate level of permethrin resistance in KL, the direct comparison of permethrin resistant mosquitoes from KL to unexposed mosquitoes from the same location very successfully detected the genes associated with permethrin resistance in this study. This is different from previous studies, in which attempts using such R-C comparison have not been too successful, notably in malaria vectors such as *An. funestus*^24^ or *An. gambiae*^25^. This is mainly because resistance level was already high in the population and the phenotypic difference between the resistant mosquitoes after exposure (R) was minimal than that of control non-exposed population.

The presence of a reduced cuticle penetration mechanism for permethrin resistance was suggested by the fact that several probes belonging to cuticular protein genes were consistently over-expressed in the KL R-C comparison. The possible role of reduced penetration was further supported because of consistency of the expression of these probes but also their higher FC with the highest over-expressed gene been always a cuticle protein (the transcript Aalb_oocyte_GH79BIP02H77ZJ with FC of 77.8; Aalb_oocyte_rep_c16319 with FC of 33.7). Over-expression of cuticular protein genes has previously been reported in several studies on insecticide resistance mechanisms such as in *An. gambiae*^21^,^26^ or in *An. funestus*^24^. However, the level of fold change observed in this *Ae. albopictus* population far exceeds that observed in these other mosquito species suggesting a more preeminent role for the reduced penetration mechanism in this population. Reduced penetration through cuticular thickening has also been previously suggested as the cause of pyrethroid resistance in the malaria vector *An. funestus,* through a cuticle thickening^27^ and the aphid *Myzus persicae*^28^.

Even though cuticular protein genes were highly over-expressed in the permethrin resistant comparison, cytochrome P450 genes were by far the gene family most abundantly over-expressed suggesting that these genes play a key role in the permethrin resistance in *Ae. albopictus* in KL. Probes from several transcripts showing the best BLAST hits to either *CYP6P12* in *Ae. aegypti* or to *CYP6P4* in *An. gambiae* were consistently the most over-expressed. Because *CYP6P12* and *CYP6P4* are orthologous genes, it is very likely that all these *Ae. albopictus* transcripts belong to the same gene, ortholog of *CYP6P12* and *CYP6P4* as supported by recent draft genome of *Ae. albopictus*^20^. It is also possible that these transcripts could belong to duplicated version of these genes as observed in *An. funestus* where the *CYP6P4* is duplicated in two copies *CYP6P4a* and *CYP6P4b*^29^. However, the involvement of the *CYP6P4* ortholog in permethrin resistance in *Ae. albopictus* will be similar to recent reports that *CYP6P4* was involved in permethrin resistance in a population of the malaria vector *An. arabiensis* in Chad^30^. *CYP6P4* has also been associated with pyrethroid resistance in *An. gambiae*^25^ and in *An. funestus*^24^,^29^. The involvement of the *CYP6P4* ortholog in the permethrin resistance in the KL population of *Ae. albopictus* is further supported by the lower expression of this gene in the C-S comparison while it is highly over-expressed in the R-C and R-S comparison. Furthermore, the transgenic expression of *CYP6P12* in Drosophila flies demonstrated that the over-transcription of this gene alone was sufficient to confer pyrethroid resistance. However, a significant difference was observed between type I and II pyrethroids as the flies expressing *CYP6P12* showed a greater mortality than control flies for type I pyrethroids, whereas it was the opposite for type II pyrethroid deltamethrin. This is consistent with the *in silico* analysis in which enzymatic activity was predicted to differ between deltamethrin and permethrin due to differences in binding position. This difference in the mortality profile of transgenic flies toward insecticides of the same class could be due to the toxicity of the intermediate metabolites generated from the detoxification of type I pyrethroids (permethrin or bifenthrin) possibly in the absence of suitable Phase II detoxification enzymes in the flies whereas such metabolites for type II are probably further detoxify by the flies. Indeed, it is known that phase I reactions catalysed by cytochrome P450 enzymes can generate reactive molecules, which is often more effective and in some cases more toxic than the parent product. And unless an efficient phase II metabolism system is in place, such intermediate metabolite can react with and cause damage to proteins, RNA and DNA with the cells^31^,^32^. It is possible that the flies expressing CYP6P12 did not have the required phase II system to deal with the generated metabolites from type I pyrethroids explaining the higher mortality observed than in control flies. Further metabolomics studies will established the nature of these metabolites. However, other technical and biological effects may also explain this unexpected result. First, a different P450 or detoxification enzyme may be responsible for permethrin resistance in the KL population with CYP6P12 being rather involved in deltamethrin resistance but still associated with permethrin resistance due to multi-resistance phenotype carried by individuals composing the KL strain. Second, it is possible that a different CYP6P12 variant than the one used for transgenesis may have the capacity to metabolize permethrin since allelic variation of P450 genes can significantly impact their metabolic ability to confer insecticide resistance as recently observed for the duplicated *CYP6P9a* and *CYP6P9b* in the African malaria vector *An. funestus*^33^. Third, the tissue-dependant over-expression pattern obtained in *Drosophila* under a generalist promoter could be different to that of the mosquito which may explain differences obtained between the two biological system (including distinct male and female effects described with bendiocarb).

Overall, no CYP9 P450 was up-regulated in the R-C comparison supporting that contrary to *Ae. aegypti*^18^,^34^ genes from this class play no or little role in pyrethroid resistance in *Ae. albopictus*.

### 2-Metabolic resistance mechanism is driving resistance in *Ae. albopictus* in Malaysia

The genome-wide transcriptional analyses carried out using microarray provided evidence that the metabolic resistance was the main mechanism conferring resistance to insecticides in *Ae. albopictus* across Malaysia. This is supported by the over-expression of many genes belonging to detoxification gene families across the three Malaysian locations and the absence of *kdr* mutations in this species across Malaysia^14^. The most preeminent detoxification gene family was again the cytochrome P450 genes which were the only detoxification family commonly over-expressed in the three locations. Among these cytochrome P450s, several transcripts of *the Ae. albopictus* gene *CYP6N3* were the most over-expressed from the list of commonly expressed genes in the three locations or only between two locations. *CYP6N3* is the ortholog of *CYP6N1* which has been shown to be over-expressed in resistant populations of the African malaria vector *An. funestus*^24^. The higher over-expression of *CYP6N3* in PG which is fully susceptible to pyrethroids suggests that this gene could be rather conferring resistance to other insecticides such as bendiocarb which is the main insecticide for which the PG population is resistant to. This is further supported by a recent study showing that *CYP6N3* was the most over-expressed P450 in an *Ae. albopictus* strain from Greece resistant to the organophosphate temephos^35^. Furthermore, the lower expression of the *CYP6N3* in the permethrin R-C comparison in KL in contrast to the C-S comparison suggests that *CYP6N3* is not involved in permethrin resistance in Malaysia.

Overall, several P450 genes belonging to the CYP6 family were over-expressed in the C-S comparisons across Malaysia including *CYP6N3*, *CYP6P12*, *CYP6Z6* and *CYP6AG6,* as also reported recently for another *Ae. albopictus* strain from Greece^35^, while only very few cytochrome P450s from the CYP9 family were over-expressed. This is in strong contrast to the other dengue vector *Ae. aegypti* for which metabolic resistance is driven mainly by cytochrome P450s belonging to the CYP9 family^10^,^18^,^34^,^36^. The pre-eminence of CYP6 family in insecticide resistance in *Ae. albopictus* is rather similar to patterns observed for *Anopheles* mosquitoes such as *An. gambiae* and *An. funestus*^24^,^37^,^38^. This difference between *Ae. albopictus* and *Ae. aegypti* suggests that the speciation between these two species is extensive and resulting to significant differences in their metabolic responses to xenobiotic-related stress.

A surprising observation in this study was the highest over-expression obtained for the three C-S comparisons for probes from the antimicrobial peptide halotricin a glycine rich repeat protein (GRRP). The potential association of this gene with insecticide resistance was further supported as it is also significantly over-expressed in the comparison between the permethrin resistant and the control population from KL (R-C) but also the permethrin resistant and the susceptible VCRU strain (R-S). Over-expression of immune response genes in insecticide resistant mosquito strains has previously been reported notably in *An. gambiae* when the defensin and cecropin genes were found up-regulated in permethrin (RSP strain) and DDT (ZANU) resistant strains^26^. The over-expression of genes belonging to other gene families such as glutathione-s-transferase, aldehyde oxidase, heat shock protein or short chain dehydrogenases has been commonly reported in other studies on mechanisms of metabolic resistance in various insects^24^,^26^,^34^,^38^.

### Absence of signature of selective sweep around *CYP6N3* and *CYP6P12*

Despite the high and consistent over-expression of *CYP6N3*, no signature of selective sweep was observed, as the gene remains highly polymorphic across Malaysia. This is similar to cases observed for other pyrethroid-metabolising P450 genes in malaria vectors such as *An. funestus* for *CYP6M7*^39^ or in *An. gambiae* for C*YP6M2* and *CYP6P3*^30^,^40^. This absence of selection on *CYP6N3* suggests that the major genetic factor conferring resistance through *CYP6N3* is probably located in a trans-acting regulatory loci which remains unknown rather than in the coding sequence. However, because several copies of this gene have been described^35^, future polymorphism analysis after cloning of CYP6N3 could be more informative in assessing the presence of selection. No clear evidence of a selection was also found on *CYP6P12,* although, because of the haplotype clustering and the lower polymorphism in some locations it cannot be ruled out that increased pyrethroid selection pressure will not lead in the future to such signatures of selection, as observed in other P450 genes such as *CYP6P9a* and *CYP6P9b*^24^ in *An. funestus* or *CYP6G1* in *Drosophila*^41^.

## Conclusion

This study has shown that insecticide resistance in *kdr*-free *Ae. albopictus* populations in Malaysia is driven by metabolic resistance, mainly under the control of cytochrome P450s, particularly *CYP6N3* and *CYP6P12*. However, a reduced penetration mechanism may also be involved against pyrethroids. If control of *Ae. albopictus* is still effective by using pyrethroids because of the relative susceptibility to this insecticide class in most populations, the presence of resistance in KL, caused mainly by *CYP6P12*, should be a concern. Therefore, suitable resistance management strategies should be implemented across Malaysia before the issue of resistance becomes worse and leads to control intervention failure.

## Materials and Methods

### Mosquito samples

*Ae. albopictus* mosquitoes were collected using ovitraps in July and August 2010 in four states, namely: Penang (Northwest), Kota Bharu (Northeast), Kuala Lumpur (Central) and Johor Bharu (South) in Malaysia as previously described^14^. Old tyres, flower pots, tree holes and containers that held water were also inspected for larvae. The larvae were reared at the Vector Control Research Unit (VCRU) in Penang (Malaysia) as recently described^14^. Adult *Ae. albopictus* were then blood fed and induced to lay eggs on filter papers that were later dried and shipped to the Liverpool School of Tropical Medicine under the LSTM import license from DEFRA. The egg batches were then hatched in the insectary in water supplemented with hay infusion solution. Larvae were reared as above and the adults were maintained on 10% sucrose solution and kept at a room temperature of 27 ± 2 °C with relative humidity of 70 ± 10%.

### Susceptibility patterns of *Ae. albopictus* across Malaysia

Data on resistance profile of the four *Ae. albopictus* populations in Malaysia have previously been described^14^. Briefly, populations *of Ae. albopictus* were fully susceptible to pyrethroids except in Kuala Lumpur where resistance was observed to permethrin and to deltamethrin (87% and 89% mortality respectively). High resistance levels against DDT were recorded in Kuala Lumpur and Kota Bharu, whereas a near full susceptibility was observed in Penang. High resistance levels were observed against bendiocarb except in Kota Bharu (93% mortality). Resistance to the organophosphate malathion was observed in the populations of Kuala Lumpur and Johor Bharu while full susceptibility was observed in Kota Bharu. The VCRU laboratory strain was used as the susceptible strain as it exhibits 100% mortality against all tested insecticides.

### Investigating metabolic resistance using microarray

A genome-wide transcription profiling was carried out to detect the sets of genes differentially expressed in relation to resistance phenotypes and possibly responsible for the metabolic resistance to insecticides in *Ae. albopictus* populations throughout Malaysia. The microarray hybridization for *Ae. albopictus* was done using a newly designed 8 × 60 k Agilent *Ae. albopictus* chip (A-MTAB-581). The chip contains 11500 *Ae. albopictus* RNAseq Expressed Sequence Tags (ESTs) from oocyst (2 probes/EST)^19^, *Ae. albopictus* transcripts from detoxification genes including cytochrome P450s from Genbank (3 probes for each) and 18600 *Ae. aegypti* transcripts (2 probes for each), to take advantage of the gene sequence conservation between these two species^19^.

Total RNA were extracted from 3 replicates of pools of 10 adult female mosquitoes not exposed to insecticide [Control (C)] from all four locations, permethrin resistant from Kuala Lumpur (KL) (alive after 1h exposure to permethrin) and from a susceptible strain from Malaysia (VCRU) using the Arcturus^®^ Picopure RNA Extraction kit (Life Technologies, California, USA), following the manufacturer’s protocol. Only samples from Penang (PG), KL and Johor Bharu (JB) were used in the microarray as the Khota Bharu (KB) samples only presented a low resistance level compared to other strains. Quality and quantity of RNA were assessed by using the Nanodrop ND-1000 (Thermo Scientific, Delaware, USA) and the Agilent 2100 Bioanalyzer (Agilent Technologies, California, USA). 100 ng of each RNA sample were amplified and labeled using the two-colour low input Quick Amp Labelling kit (Agilent Technologies). Labeled cRNAs were hybridized to the arrays for 17 h at 65 °C according to the manufacturer’s protocol. Two different experiments were designed to characterise the resistance mechanisms. The first design (Fig. 1b) compared control or non-exposed samples (C) from Penang, Kuala Lumpur and Johor Bharu against the susceptible VCRU lab strain with the aim to detect most of the genes associated with metabolic resistance to all insecticides in three locations. The second experiment was designed to further investigate mechanisms of permethrin resistance observed in Kuala Lumpur through three different comparisons; KL permethrin-resistant (alive after 1 h exposure) against VCRU susceptible lab strain (R-S), KL non-exposed against VCRU (C-S) and KL permethrin-resistant against KL non-exposed (R-C) (Fig. 1c). This triangular design has been successfully used to detect pyrethroid resistance genes in the malaria vector *An. funestus*^24^. Five replicates (including three biological replicates and two dye swaps) were performed for each comparison as previously successfully done in other species to detect the resistance genes^24^,^38^.

Microarray data were analysed using the Genespring GX 12.0 software (Agilent Technologies, US). Mean expression ratios were assessed using a t-test against zero with a multiple testing correction (Benjamini-Hochberg false discovery rate). Genes showing both t-test p-values less than 0.01 and a fold change value of 2 were considered significantly differentially transcribed between the two samples compared.

### Validation of candidate genes using qRT-PCR

The top candidate genes that were significantly differentially expressed from the microarray analysis were validated using qRT-PCR. Total RNA from 3 replicates of samples PG, KL, JB, permethrin resistant from KL and VCRU were used to synthesize the cDNA using Superscript III (Invitrogen) with oligo-dT20 and RNase H according to the manufacturer’s instructions. In addition, expression of the genes was also investigated in the samples from KB that was not used in the microarray to access their potential role in this population. The primers used are listed in Table S9. Standard curves for each gene were generated using a serial dilution of cDNA allowing to assess PCR efficiency and quantitative differences between samples. qRT-PCR amplification was performed as described previously^24^,^38^. The relative expression level and Fold Change (FC) of each target gene field sample relative to the susceptible VCRU (S) were calculated according to the 2^−ΔΔCT^ method incorporating the PCR efficiency^42^ after normalization with the housekeeping genes ribosomal protein S7 (*RSP7*; AAEL009496-RA) and Tubulin (AAEL009496-RA). A two-sample t-test was used to assess the statistical significance of the results between samples.

### Functional validation of the role of *CYP6P12* using transgenic expression in *Drosophila melanogaster*

#### Construction of transgenic *D. melanogaster* strain

In order to determine if the up-regulation of the candidate resistance gene *CYP6P12* is sufficient to independently confer resistance to pyrethroid insecticides, transgenic *D. melanogaster* expressing *CYP6P12* were generated using the GAL4/UAS system. The transgenic strain was constructed following the protocol previously described^24^,^43^. Briefly, the full length of the *CYP6P12* gene was amplified using Kuala Lumpur cDNAs with the Phusion High-Fidelity DNA Polymerase (Thermo Scientific) and cloned into the pJET1.2/blunt cloning vector (Thermo Scientific). The primers are: Alb_6P4_F_BgLII: ATA GAT CTA TGT TAG CTT ATT TAT TGG CGG; Alb_6P4_R_XBaI: TCT CTA GAT CAT ATC TTA TCG TAC CGA. The most predominant clone from KL was selected to construct transgenic flies and cloned into the pUASattB vector using primers containing restriction sites for *BglII* and *Xba*I. Using the PhiC31 system, clones were transformed into the germ line of a *D. melanogaster* strain carrying the attP40 docking site on chromosome 2 [“y^1^w^67c23^; P attP40”, “1;2”] by Genetic Services (MA, USA) to generate the transgenic line UAS-CYP6P12.

#### Expression of *CYP6P12* using the GAL4/UAS system

An ubiquitous expression of the transgene *CYP6P12* in adult F_1_ progeny (experimental group) was generated by crossing virgin females from the driver strain Act5C-GAL4 [“y[1] w[*]; P(Act5C-GAL4-w)E1/CyO”,“1;2”] (Bloomington Stock Center, IN, USA) with males homozygote UAS-CYP6P12. Similarly, adult F_1_ control progeny (control group) with the same genetic background as the experimental group but without expression of *CYP6P12* were obtained by crossing virgin females from the driver strain Act5C-GAL4 and UAS recipient line males (which do not carry the pUASattb-CYP6P12 insertion).

#### *Drosophila* bioassays

The ability of the *CYP6P12* to confer resistance to pyrethroids and other insecticides was assessed by performing insecticide bioassays with experimental and control F_1_ *D. melanogaster* females. Two to five day old flies post-eclosion females were used for bioassays with 0.15% deltamethrin, 2% permethrin, 0.2%bifenthrin and 2% etofenprox. The carbamate insecticide bendiocarb was also tested at concentrations of 0.01%. The insecticide-impregnated filter papers were prepared in acetone and Dow Corning 556 Silicone Fluid (BHD/Merck, Germany). These papers were rolled and introduced into 45 cc plastic vials to cover the entire wall. The vials were plugged with cotton soaked in 5% sucrose. Then, 20–25 flies were placed in each vial, and the mortality plus knockdown was scored after 1 h, 2 h, 3 h, 6 h and 24 h (and in some cases 12 and 48 h) of exposure to the insecticide. For all assays, at least 6 replicates were performed. The mortality rates between the experimental (*GAL4-actin* x *UAS-6P4*) and the control (*GAL4-actin* x *UAS-NO*) lines were used to determine the role of *CYP6P12* in insecticide metabolism using the students’ t-test statistical analysis.

### Analysis of polymorphism patterns of candidate genes

The full-length CYP6P12 and CYP6N3 were amplified, cloned and sequenced as described above using the same cDNA synthesised for qRT-PCR with the Phusion polymerase. Polymorphic positions were detected through manual analysis of sequence traces using BioEdit and as sequence differences in multiple alignments using ClustalW^44^. DnaSP 5.1^45^ was used to assess genetic parameters of each gene, such as nucleotide diversity (π) and haplotype diversities. A maximum likelihood tree of the haplotypes for each genes was constructed using MEGA 6.0^46^ using the Tamura-Nei model with 500 bootstraps.

### *In silico* modelling and docking of 3D Insecticide

In order to predict if pyrethroids and other insecticides bind productively in the active site of *CYP6P12*, a molecular docking simulation was carried out, binding energy and conformations analysed. 3D homology models of this P450 was created based on the crystal structure of human CYP3A4 (PDB:1TQN)^47^ using the MODELLER^48^. CYP3A4 shares 33% identity for CYP6P12. Virtual datasets of ligand insecticides: permethrin (ZINC01850374), deltamethrin (ZINC01997854), bendiocarb (ZINC02015426) and DDT (ZINC01530011) were retrieved from the library of ZINC^12^ (https://zinc.docking.org/) database in MOL2 format^49^.

Docking simulations were carried out using the *CLC bio* Drug Discovery Workbench 2.0 (http://www.clcbio.com/products/clc-drug-discovery-workbench/) with binding site set as a sphere centred above the heme iron and covering 20Å radius. For each ligand, 50 binding poses were generated and sorted according to hybrid PLANTS_PLP_ score^50^ and conformation in the protein’s active site. Figures were prepared using the PYMOL 1.7^51^.

## Additional Information

**How to cite this article**: Ishak, I. H. *et al.* The Cytochrome P450 gene *CYP6P12* confers pyrethroid resistance in *kdr*-free Malaysian populations of the dengue vector *Aedes albopictus. Sci. Rep.* **6**, 24707; doi: 10.1038/srep24707 (2016).

## Supplementary Material

Supplementary Information

## Figures and Tables

**Table 1:** Detoxification transcripts up regulated across Malaysia when comparing non exposed mosquitoes to VCRU susceptible strain (C-S).

Probe name	Transcript-ID	Absolute – FC			Description
		Penang	Johor Bharu	Kuala Lumpur	
**Detoxification**					
CUST_90_PI427639958	HQ621851.1 (CYP6N9 in *Ae. aegypti*)	11.1	3.4	3.8	Cytochrome P450
CUST_17858_PI427639955	Aalb_oocyte_rep_c24780 (CYP9AE1 in *Ae. aegypti*)	6.3	4.0	2.2	Cytochrome p450
CUST_15781_PI427639947	AAEL006992-RA (CYP6AG6 in *Ae. aegypti*)	3.0	2.8	9.6	Cytochrome p450
CUST_115_PI427639958	JF317342.1 (CYP6N3)	25.1		7.8	Cytochrome p450
CUST_118_PI427639958	JF317340.1 (CYP6N3)	23.1		7.9	Cytochrome p450
CUST_130_PI427639958	JF317341.1 (CYP6N3)	22.7		7.5	Cytochrome p450
CUST_18637_PI427639955	Aalb_oocyte_rep_c28874 (CYP6AG6 in *Ae. aegypti*)	2.9		2.8	cytochrome p450
CUST_13399_PI427639947	AAEL009656-RA (CYP6AL3 in *Ae. aegypti*)	2.0		2.9	cytochrome P450
CUST_17859_PI427639955	Aalb_oocyte_rep_c24780 (CYP9AE1 in *Ae. aegypti*)	6.7	4.9		cytochrome p450
CUST_35612_PI427639947	AAEL015432-RA	7.0	3.5		trypsin, putative
CUST_1663_PI427639955	Aalb_oocyte_rep_c11155 (GSTT3 in *Ae. aegypti*)	6.5	10.2		glutathione-s-transferase gst
CUST_19418_PI427639955	Aalb_oocyte_rep_c4101	5.4	11.0		abc transporter
CUST_263_PI427639958	AF284783.1 (CYP6N3v4)	5.3	5.6		Cytochrome p450
CUST_21111_PI427639955	Aalb_oocyte_GH79BIP02HN8AL	5.0	4.6		atp-binding cassette transporter
CUST_7878_PI427639947	AAEL005491-RA	4.6	5.3		ABC transporter
CUST_21101_PI427639955	Aalb_oocyte_c13494 (CYP9M6 in *Ae. aegypti*)	4.6	6.1		cytochrome p450
CUST_21328_PI427639955	Aalb_oocyte_c30071 (GSTD5 in *Ae. aegypti*)	4.4	13.7		glutathione s-transferase
CUST_9941_PI427639955	Aalb_oocyte_GH79BIP02GBWB9 (CYP6P12 in *Ae.aegypti*)	3.5	10.5		cytochrome p450
CUST_92_PI427639958	HQ621849.1 (CYP6P12 in *Ae.aegypti*)	3.3	3.7		Cytochrome p450
CUST_857_PI427639955	Aalb_oocyte_rep_c13705 (CYP6ZB1 in *Ae. aegypti* and CYP6P4 in *An. gambiae*)	2.8	4.6		cytochrome p450
CUST_87_PI427639958	HQ621853.1 (CYP6N3)	2.6	2.1		Cytochrome p450
CUST_981_PI427639955	Aalb_oocyte_rep_c46923 (GSTE3 in *Ae.aegypti*)	2.6	3.9		glutathione-s-transferase gst
CUST_135_PI427639958	JF317339.1 (CYP6P4 in *An. gambiae*)	2.5	3.0		Cytochrome p450
CUST_9720_PI427639955	Aalb_oocyte_GIK0NFC01CWBYU	2.4	2.3		carboxylesterase
CUST_21999_PI427639955	Aalb_oocyte_rep_c13281 (CYP6Z8 in *Ae. aegypti*)	2.4	3.9		cytochrome p450
CUST_9506_PI427639955	Aalb_oocyte_rep_c6282	2.3	2.2		atp-binding cassette sub-family
CUST_982_PI427639955	Aalb_oocyte_rep_c46923 (GSTE3 in *Ae.aegypti*)	2.3	5.3		glutathione-s-transferase gst
CUST_22316_PI427639947	AAEL009123-RA (CYP6Z6 in *Ae.aegypti*)	2.3	4.7		cytochrome P450
CUST_134_PI427639958	JF317339.1 (CYP6P4 in *An. gambiae*)	2.3	2.7		Cytochrome p450
CUST_858_PI427639955	Aalb_oocyte_rep_c13705 (CYP6ZB1 in *Ae. aegypti* and CYP6P4 in *An. gambiae*)	2.3	4.9		cytochrome p450
CUST_122_PI427639958	JF317338.1 (CYP6P4 in *An. gambiae*)	2.0	2.1		Cytochrome p450
