# Supplementary material for: The Cytochrome P450 gene CYP6P12 confers pyrethroid resistance in kdr-free Malaysian populations of the dengue vector Aedes albopictus
Source: Sci Rep. 2016 Apr 20;6:24707. doi: 10.1038/srep24707 (PMC4837359; doi:10.1038/srep24707)
Supplement: Supplementary Information [file srep24707-s1.pdf]

The Cytochrome P450 gene *CYP6P12* confers pyrethroid resistance in *kdr*-free Malaysian populations of the dengue vector *Aedes albopictus*

Intan H. Ishak[1,2#], Jacob M. Riveron[1#], Sulaiman S. Ibrahim[1], Rob Stott[1], Joshua Longbottom[1], Helen Irving[1] and Charles S. Wondji[1*]

[1] Department of Vector Biology, Liverpool School of Tropical Medicine, Pembroke Place, Liverpool L3 5QA, United Kingdom

[2] School of Biological Sciences, Universiti Sains Malaysia, 11800 Penang, Malaysia

[*] to whom reprint requests should be addressed. Email: charles.wondji@lstmed.ac.uk

# contributed equally

Table S1: Transcripts from detoxification genes and genes associated with permethrin resistance up-regulated in various comparisons (R-C, R-S and C-S) with P<0.01 and Fold-change >2 in Kuala Lumpur

Probe Names	Transcripts	R-C	R-S	C-S	Description
CUST_18637_PI427639955	Aalb_oocyte_rep_c28874 (as CYP6AG6 in *Ae. aegypti*)	2.9	3.9	2.8	cytochrome p450
CUST_30823_PI427639947	AAEL017536-RA	4.7	8.9	2.7	Holotricin, Glycine Rich Repeat Protein (GRRP), Anti-Microbial Peptide.
CUST_2213_PI427639955	Aalb_oocyte_c29950 (AAEL0010384)	3.7	3.3		aldehyde oxidase
CUST_21698_PI427639955	Aalb_oocyte_rep_c21955 (as AAEL013543)	9.1	7.7		alpha-esterase
CUST_35289_PI427639947	AAEL015264-RA	2.1	3.2		alpha-esterase
CUST_9941_PI427639955	Aalb_oocyte_GH79BIP02GBWB9 (as CYP6P12 in *Ae.aegypti*)	31.8	23.3		cytochrome p450
CUST_121_PI427639958	JF317338.1 (as CYP6P4 in *An. gambiae*)	4.9	3.8		Aedes albopictus clone 22v1 cytochrome P450
CUST_135_PI427639958	JF317339.1(as CYP6P4 in *An. gambiae*)	11.2	8.2		Aedes albopictus clone 22v2 cytochrome P450
CUST_92_PI427639958	HQ621849.1(as CYP6P12 in *Ae.aegypti*)	11.2	10.9		Aedes albopictus isolate P18 cytochrome P450
CUST_7196_PI427639955	Aalb_oocyte_c5450	2.6	2.7		cytochrome p450
CUST_8072_PI427639955	Aalb_oocyte_GIK0NFC01EFN86 GIK0NFC01EFN86 (as CYP6ZB1 in *Ae. aegypti* and CYP6P4 *An. gambiae*)	9.3	10.2		cytochrome p450
CUST_21999_PI427639955	Aalb_oocyte_rep_c13281	6.7	4.8		cytochrome p450
CUST_858_PI427639955	Aalb_oocyte_rep_c13705 (as CYP6ZB1 in *Ae. aegypti* and CYP6P4 in *An. gambiae*)	5.6	5.9		cytochrome p450
CUST_16144_PI427639955	Aalb_oocyte_rep_c20820	3.3	2.8		cytochrome p450
CUST_41_PI427639955	Aalb_oocyte_rep_c27556	4.1	2.5		cytochrome p450
CUST_1521_PI427639955	Aalb_oocyte_rep_c5392	3.0	2.3		cytochrome p450
CUST_22585_PI427639955	Aalb_oocyte_rep_c925	4.2	4.0		cytochrome p450
CUST_12011_PI427639947	AAEL004054-RA	3.1	2.5		cytochrome P450
CUST_12531_PI427639947	AAEL006824-RA	2.3	2.5		cytochrome P450

CUST_17940_PI427639947	AAEL008345-RA	2.5	2.6	cytochrome P450
CUST_20967_PI427639947	AAEL009131-RA	4.9	3.1	cytochrome P450
CUST_27662_PI427639947	AAEL015370-RA	3.7	2.8	cytochrome P450
CUST_27886_PI427639947	AAEL011741-RB (GSTS1 in *Ae. aegypti*)	20.2	3.5	glutathione-s-transferase
CUST_1663_PI427639955	Aalb_oocyte_rep_c11155 (GSTT3 in *Ae. aegypti*)	16.5	16.4	glutathione-s-transferase gst
CUST_8352_PI427639947	AAEL004229-RA (GSTT4 in *Ae. aegypti*)	5.1	4.0	glutathione-s-transferase
CUST_20871_PI427639955	Aalb_oocyte_rep_c4004 (GSTT4 in *Ae. aegypti*)	4.3	3.7	glutathione-s-transferase gst
CUST_14233_PI427639955	Aalb_oocyte_rep_c7700 (GSTS1 in *Ae. aegypti*)	3.7	2.1	glutathione s-transferase
CUST_29825_PI427639947	AAEL014863-RB	8.4	3.0	glycogenin
CUST_26891_PI427639947	AAEL014863-RD	8.7	3.0	glycogenin
CUST_33999_PI427639947	AAEL014863-RE	8.6	3.0	glycogenin
CUST_32777_PI427639947	AAEL014279-RA	60.7	47.6	glycosyltransferase
CUST_4561_PI427639955	Aalb_oocyte_rep_c16438	6.2	2.6	heat shock protein
CUST_15623_PI427639955	Aalb_oocyte_rep_c20783	6.7	3.1	heat shock protein
CUST_32384_PI427639947	AAEL015090-RA	4.6	2.4	heat shock protein
CUST_30971_PI427639947	AAEL012481-RA	4.5	3.2	heme peroxidase
CUST_19861_PI427639955	Aalb_oocyte_rep_c14000	9.6	5.1	lethal essential for life l2efl
CUST_23050_PI427639947	AAEL010659-RA	5.1	2.5	lethal(2)essential for life protein, l2efl
CUST_28821_PI427639947	AAEL013343-RA	4.8	2.3	lethal(2)essential for life protein, l2efl
CUST_10354_PI427639955	Aalb_oocyte_rep_c25391	4.3	2.4	odorant-binding protein
CUST_24258_PI427639947	AAEL007603-RA	4.4	3.1	odorant-binding protein 56a, putative
CUST_17606_PI427639955	Aalb_oocyte_rep_c10533	3.5	4.9	odorant-binding protein 99a
CUST_1589_PI427639955	Aalb_oocyte_GH79BIP02H77ZJ (as AGAP008449 or AAEL002181)	77.3	7.0	cuticle protein
CUST_7294_PI427639947	AAEL003049-RA	56.2	14.1	pupal cuticle protein 78E, putative
CUST_4199_PI427639955	Aalb_oocyte_rep_c33277 (As AAEL003259)	37.3	11.1	pupal cuticle protein
CUST_21736_PI427639955	Aalb_oocyte_rep_c16319 (As AAEL003235)	33.8	13.9	larval cuticle protein lcp-30

CUST_4058_PI427639947	AAEL002181-RA	28.8	5.3		cuticle protein, putative
CUST_2672_PI427639955	Aalb_oocyte_rep_c18373 (As AAEL013515)	27.2	14.7		pupal cuticle
CUST_12759_PI427639947	AAEL008285-RA	15.6	4.4		pupal cuticle protein, putative
CUST_20107_PI427639955	Aalb_oocyte_rep_c34982 (As AAEL011045)	9.0	5.6		pupal cuticle protein
CUST_19091_PI427639947	AAEL011045-RA	6.7	3.7		pupal cuticle protein, putative
CUST_21803_PI427639947	AAEL009791-RA	4.4	3.5		cuticle protein, putative
CUST_13900_PI427639955	Aalb_oocyte_rep_c24663 (As AAEL009801)	3.2	3.1		cuticle
CUST_20739_PI427639947	AAEL008227-RA	3.3	2.5		short-chain dehydrogenase
CUST_29165_PI427639947	AAEL012702-RA	2.0	2.7		ABC sub-family A member 3
CUST_118_PI427639958	JF317340.1(CYP6N3)		6.0	7.8	cytochrome p450
CUST_115_PI427639958	JF317342.1(CYP6N3)		5.7	7.8	cytochrome p450
CUST_130_PI427639958	JF317341.1(CYP6N3)		5.8	7.4	cytochrome p450
CUST_90_PI427639958	HQ621851.1 (CYP6N9 in *Ae. aegypti*)		4.2	3.8	cytochrome p450
CUST_15854_PI427639947	AAEL008397-RA		2.4	2.5	glutathione peroxidase

Table S2: Top 20 commonly down-regulated probes in *Ae. albopictus* in Kuala Lumpur R-C, R-S and C-S in comparison with susceptible VCRU strain. FC = fold change (p = 0.01).

Probe name	Gene-ID	Absolute – FC			Description
		R-C	R-S	C-S	
CUST_48_PI427639955	Aalb_oocyte_rep_c14495	7.3	41.6	6.4	serine threonine-protein kinase rio2
CUST_664_PI427639955	Aalb_oocyte_rep_c171	12.6	27.4	4.6	4-nitrophenylphosphatase
CUST_20261_PI427639947	AAEL007097-RA	9.7	25.7	4.6	4-nitrophenylphosphatase
CUST_663_PI427639955	Aalb_oocyte_rep_c171	12.2	27.8	4.5	4-nitrophenylphosphatase
CUST_18445_PI427639955	Aalb_oocyte_GH79BIP02I1W9U	9.6	20.4	4.2	---NA---
CUST_18444_PI427639955	Aalb_oocyte_GH79BIP02I1W9U	8.6	16.1	4.2	---NA---
CUST_19484_PI427639955	Aalb_oocyte_GH79BIP01AY0KB	7.3	12.4	4.2	lethal essential for life l2efl
CUST_9025_PI427639955	Aalb_oocyte_GH79BIP01A3RBT	4.8	7.6	3.8	---NA---
CUST_22690_PI427639955	Aalb_oocyte_rep_c838	6.1	13.8	3.6	lethal essential for life l2efl
CUST_22691_PI427639955	Aalb_oocyte_rep_c838	5.9	12.2	3.6	lethal essential for life l2efl
CUST_3019_PI427639955	Aalb_oocyte_rep_c21768	5.2	7.5	2.6	cysteine-rich venom
CUST_16355_PI427639955	Aalb_oocyte_rep_c13318	7.0	8.5	2.6	---NA---
CUST_16354_PI427639955	Aalb_oocyte_rep_c13318	5.6	8.5	2.5	---NA---
CUST_24391_PI427639947	AAEL010094-RA	3.5	6.0	2.4	cyclin b
CUST_6286_PI427639955	Aalb_oocyte_rep_c11762	4.7	2.6	2.2	isoform a
CUST_6665_PI427639947	AAEL005495-RA	2.5	2.8	2.1	phospholipid-transporting ATPase 1 (aminophospholipid flippase 1)
CUST_22063_PI427639955	Aalb_oocyte_rep_c13096	3.0	6.7	2.1	hypothetical protein AaeL_AAEL004104 [Aedes aegypti]
CUST_4104_PI427639947	AAEL002565-RA	8.0	2.9	2.1	titin
CUST_14783_PI427639955	Aalb_oocyte_c31200	5.9	2.1	2.1	isoform g
CUST_20890_PI427639955	Aalb_oocyte_rep_c10794	2.6	4.6	2.1	leucine rich protein

Table S3: Main gene families commonly up-regulated in all three locations in comparison with susceptible VCRU strain. FC = fold change.

Probe name	Gene-ID	Absolute – FC			Description
		Penang	Johor Bharu	Kuala Lumpur	
**Detoxification**					
CUST_90_PI427639958	HQ621851.1 (as CYP6N9 in *Ae. aegypti*)	11.06717	3.390716	3.80548	Cytochrome P450
CUST_17858_PI427639955	Aalb_oocyte_rep_c24780 (as CYP9AE1 in *Ae. aegypti*)	6.349737	4.006419	2.186192	Cytochrome p450
CUST_15781_PI427639947	AAEL006992-RA (as CYP6AG6 in *Ae. aegypti* & CYP6AG1 in *An. gambiae*)	3.014005	2.769197	9.60161	Cytochrome p450
**Redox/mitochondrial**					
CUST_18659_PI427639955	Aalb_oocyte_rep_c13574	3.46779	7.934232	3.256955	acetyl- mitochondrial
**Protein synthesis/metabolism**					
CUST_33850_PI427639947	AAEL014562-RA	3.267709	3.464005	2.240465	60S ribosomal protein L12
CUST_3111_PI427639955	Aalb_oocyte_rep_c35888	5.467519	6.689628	2.321785	cathepsin l
CUST_24197_PI427639947	AAEL008853-RA	3.431536	3.63372	2.979337	choline/ethanolamine kinase
CUST_9602_PI427639955	Aalb_oocyte_rep_c42656	3.936562	3.891619	2.101489	mitochondrial ribosomal protein l44
CUST_5613_PI427639955	Aalb_oocyte_rep_c21876	6.808945	10.22998	5.400242	probable ribosome biogenesis protein c16orf42-like
CUST_13353_PI427639955	Aalb_oocyte_GIK0NFC01AZEAV	8.486282	6.111363	2.1815	serine protease
CUST_12040_PI427639955	Aalb_oocyte_rep_c61861	4.325947	3.670383	2.0646	serine protease
CUST_20414_PI427639955	Aalb_oocyte_rep_c2630	3.255203	2.629312	2.35589	serine threonine-protein kinase rio2

**Lipid/carbohydrate synthesis/metabolism**					
CUST_2982_PI427639955	Aalb_oocyte_GH79BIP02FWVVV	4.160906	4.088471	2.971394	lipoprotein lipase
**Transport/ion transport**					
CUST_13058_PI427639947	AAEL008381-RA	2.248142	3.224599	2.168007	oligopeptide transporter
CUST_5895_PI427639955	Aalb_oocyte_rep_c7850	3.644206	2.972166	2.548524	sugar transporter
**Immune defence**					
CUST_30823_PI427639947	AAEL017536-RA	12.97018	12.63358	2.931466	Holotricin, Glycine Rich Repreat Protein (GRRP), Anti-Microbial Peptide.
CUST_763_PI427639955	Aalb_oocyte_rep_c27383	10.03429	6.767646	2.126202	hypothetical mtt-rich mucin
**Other**					
CUST_15452_PI427639947	AAEL009396-RA	2.8108	3.307311	2.689146	amine oxidase
CUST_11965_PI427639955	Aalb_oocyte_rep_c10601	12.65986	28.7097	3.635909	t-dirnahydrouridine synthase
CUST_21247_PI427639947	AAEL007160-RA	12.22205	12.42675	9.207074	ubiquilin 1,2
CUST_17631_PI427639955	Aalb_oocyte_GH79BIP02GTORD	6.993401	8.338801	5.054333	AGAP006143-PE [Anopheles gambiae str. PEST]
CUST_12982_PI427639955	Aalb_oocyte_rep_c61338	6.629262	4.511744	2.134098	cg31751 cg31751-pa
CUST_6496_PI427639955	Aalb_oocyte_rep_c1566	3.750916	4.260312	2.801973	cg4553 cg4553-pa
CUST_32485_PI427639947	AAEL014556-RB	2.942438	3.009454	2.273961	conserved hypothetical protein
CUST_29183_PI427639947	AAEL015053-RB	2.808157	3.227621	2.324586	conserved hypothetical protein
CUST_19802_PI427639947	AAEL006433-RA	2.561922	3.224315	2.347336	conserved hypothetical protein
CUST_7711_PI427639947	AAEL003929-RA	2.55509	8.356735	3.328963	conserved hypothetical protein
CUST_1287_PI427639947	AAEL000615-RA	2.011041	5.307753	2.189566	hypothetical protein
CUST_20050_PI427639955	Aalb_oocyte_GH79BIP01CBET4	5.582831	3.567648	2.654278	hypothetical protein AaeL_AAEL013040
CUST_3055_PI427639955	Aalb_oocyte_c20316	8.572461	2.958011	6.382831	kda secreted protein -1
CUST_4527_PI427639955	Aalb_oocyte_rep_c58107	2.195233	2.221553	2.024178	kda secreted salivary peptide

Table S4: Detoxification transcripts up-regulated in only a single location in C-S Comparison

Probe name	Transcript-ID	Absolute – FC			Description
		Penang	Johor Bharu	Kuala Lumpur	
CUST_1664_PI427639955	Aalb_oocyte_rep_c11155 (GSTT3 in *Ae. aegypti*)	9.6			glutathione-s-transferase gst theta
CUST_132_PI427639958	JF317341.1 (CYP6N3)	6.6			cytochrome p450
CUST_2672_PI427639955	Aalb_oocyte_rep_c18373	6.0			cytochrome p450
CUST_15444_PI427639955	Aalb_oocyte_rep_c11991 (CYP9J17 in *Ae. aegypti*)	6.0			cytochrome p450
CUST_2671_PI427639955	Aalb_oocyte_rep_c18373	6.0			pupal cuticle
CUST_120_PI427639958	JF317340.1 (CYP6N3)	6.0			cytochrome p450
CUST_27663_PI427639947	AAEL015370-RA (CYP4J9 in *An. gambiae*)	5.5			cytochrome P450
CUST_7355_PI427639955	Aalb_oocyte_c25640 (CYP6N11 in *Ae. aegypti*)	5.0			cytochrome p450
CUST_23124_PI427639947	AAEL009124-RA (CYP6N12 in *Ae. aegypti*)	5.0			cytochrome P450
CUST_27549_PI427639947	AAEL014019-RA (CYP4J16 in *Ae. aegypti*)	3.9			cytochrome P450
CUST_21973_PI427639955	Aalb_oocyte_rep_c21808 (CYP6P12 in *Ae. aegypti*)	3.6			cytochrome p450
CUST_35854_PI427639947	AAEL013834-RA	3.5			ATP-binding cassette transporter
CUST_133_PI427639958	JF317339.1	2.2			cytochrome p450
CUST_73_PI427639958	HQ621852.1	2.1			cytochrome p450
CUST_7990_PI427639955	Aalb_oocyte_rep_c11431	2.4			abc transporter
CUST_31953_PI427639947	AAEL014699-RA	2.9			ABC transporter
CUST_13036_PI427639947	AAEL008631-RA	2.7			ABC transporter
CUST_12693_PI427639947	AAEL008632-RA	2.2			ABC transporter
CUST_21699_PI427639955	Aalb_oocyte_rep_c21955	7.9			alpha-esterase
CUST_1980_PI427639955	Aalb_oocyte_GID1I5S02FGQN7	4.8			alpha-esterase
CUST_3172_PI427639955	Aalb_oocyte_rep_c62843	3.5			alpha-esterase
CUST_35289_PI427639947	AAEL015264-RA	4.1			alpha-esterase
CUST_7100_PI427639955	Aalb_oocyte_rep_c20306	2.5			aquaporin
CUST_3934_PI427639955	Aalb_oocyte_GIK0NFC01B79W0	2.5			atp-binding cassette sub-family
CUST_9505_PI427639955	Aalb_oocyte_rep_c6282	2.1			atp-binding cassette sub-family
CUST_26912_PI427639947	AAEL012698-RA	2.8			ATP-binding cassette sub-family A

CUST_13441_PI427639947	AAEL008384-RA	2.1	ATP-binding cassette sub-family A
CUST_20077_PI427639955	Aalb_oocyte_rep_c3955	2.4	atp-binding cassette sub-family f
CUST_21110_PI427639955	Aalb_oocyte_GH79BIP02HN8AL	2.8	atp-binding cassette transporter
CUST_35854_PI427639947	AAEL013834-RA	3.5	ATP-binding cassette transporter
CUST_24017_PI427639947	AAEL010379-RA	2.8	ATP-binding cassette transporter
CUST_21100_PI427639955	Aalb_oocyte_c13494	2.9	cytochrome p450
CUST_16309_PI427639955	Aalb_oocyte_GH79BIP01C4IX6	2.8	cytochrome p450
CUST_808_PI427639955	Aalb_oocyte_rep_c15168	2.6	cytochrome p450
CUST_21972_PI427639955	Aalb_oocyte_rep_c21808	2.4	cytochrome p450
CUST_16308_PI427639955	Aalb_oocyte_GH79BIP01C4IX6	2.4	cytochrome p450
CUST_22304_PI427639955	Aalb_oocyte_GH79BIP01DFLKN	2.2	cytochrome p450
CUST_1522_PI427639955	Aalb_oocyte_rep_c5392	2.2	cytochrome p450
CUST_17939_PI427639947	AAEL008345-RA	2.7	cytochrome P450
CUST_28407_PI427639947	AAEL014606-RA	2.7	cytochrome P450
CUST_13400_PI427639947	AAEL009656-RA	2.5	cytochrome P450
CUST_12532_PI427639947	AAEL006824-RA	2.5	cytochrome P450
CUST_8756_PI427639947	AAEL003748-RA	2.3	cytochrome P450
CUST_27114_PI427639947	AAEL012491-RA	2.2	cytochrome P450
CUST_14345_PI427639947	AAEL007024-RA	2.1	cytochrome P450
CUST_12012_PI427639947	AAEL004054-RA	2.1	cytochrome P450
CUST_17940_PI427639947	AAEL008345-RA	2.0	cytochrome P450
CUST_9395_PI427639955	Aalb_oocyte_rep_c11643	2.3	glucosyl glucuronosyl transferases
CUST_3013_PI427639947	AAEL001364-RA	2.2	glucosyl/glucuronosyl transferases
CUST_17306_PI427639955	Aalb_oocyte_GH79BIP02I2N9M	2.2	glutathione-s-transferase gst
CUST_21870_PI427639955	Aalb_oocyte_rep_c39117	2.4	glycoprotein 93
CUST_13664_PI427639955	Aalb_oocyte_GIK0NFC01DFP1Z	2.4	short-chain dehydrogenase
CUST_3925_PI427639947	AAEL001845-RA	3.0	short-chain dehydrogenase
CUST_19192_PI427639947	AAEL006224-RA	2.7	short-chain dehydrogenase
CUST_35356_PI427639947	AAEL011488-RA	2.7	short-chain dehydrogenase
CUST_9897_PI427639947	AAEL005703-RA	2.3	short-chain dehydrogenase
CUST_3994_PI427639947	AAEL002901-RA	2.1	short-chain dehydrogenase

CUST_13508_PI427639955	Aalb_oocyte_GH79BIP01CA6AP	2.0			thioredoxin family trp26
CUST_33194_PI427639947	AAEL014548-RA	4.1			thioredoxin peroxidase
CUST_27371_PI427639947	AAEL013528-RA	2.2			thioredoxin peroxidase
CUST_30368_PI427639947	AAEL013703-RA	2.3			trypsin
CUST_3159_PI427639947	AAEL001061-RB (GSTD1 in *Ae. aegypti*)		4.6		glutathionetransferase
CUST_242_PI427639958	AF284788.1 (CYP6N4)		4.0		cytochrome P450
CUST_26373_PI427639947	AAEL014614-RA (CYP9J4 in *An. gambiae*)		4.0		cytochrome P450
CUST_245_PI427639958	AF284786.1 (CYP6N4)		3.8		cytochrome P450
CUST_27548_PI427639947	AAEL014019-RA (CYP4J16 in *Ae. aegypti*)		3.7		cytochrome P450
CUST_21998_PI427639955	Aalb_oocyte_rep_c13281 (CYP6Z8 in *Ae. aegypti*)		3.7		cytochrome p450
CUST_22681_PI427639955	Aalb_oocyte_rep_c32138		3.7		aldehyde oxidase
CUST_15200_PI427639955	Aalb_oocyte_rep_c7225		3.4		short-chain dehydrogenase
CUST_8072_PI427639955	Aalb_oocyte_GIK0NFC01EFN86 (CYP6ZB1 in *Ae. aegypti* and CYP6P4 *An. gambiae*)		2.8		cytochrome p450
CUST_7991_PI427639955	Aalb_oocyte_rep_c11431		2.5		abc transporter
CUST_9872_PI427639955	Aalb_oocyte_GIK0NFC01AJOWD		2.4		cuticular protein
CUST_22585_PI427639955	Aalb_oocyte_rep_c925 (CYP9J27 in *Ae.aegypti*)		2.2		cytochrome p450
CUST_2000_PI427639955	Aalb_oocyte_rep_c15442 (CYP9J15 in *Ae.aegypti*)		2.1		cytochrome p450
CUST_18374_PI427639955	Aalb_oocyte_rep_c61320		2.0		short-chain dehydrogenase
CUST_5173_PI427639955	Aalb_oocyte_GH79BIP01BBCTN		2.0		trypsin
CUST_32539_PI427639947	AAEL012189-RA		2.0		ATP-binding cassette C1)
CUST_32777_PI427639947	AAEL014279-RA		24.2		Glycosyltransferase
CUST_10095_PI427639947	AAEL004941-RA (CYP6AK1 in *Ae. aegypti*)			2.9	cytochrome P450
CUST_22169_PI427639955	Aalb_oocyte_GH79BIP02I9E16 (CYP6AG5 in *Ae. aegypti*)			2.5	cytochrome p450
CUST_10966_PI427639955	Aalb_oocyte_rep_c403			2.3	chymotrypsin-like protein
CUST_10965_PI427639955	Aalb_oocyte_rep_c403			2.3	chymotrypsin-like protein
CUST_18636_PI427639955	Aalb_oocyte_rep_c28874 (CYP6AG6 in *Ae. aegypti*)			2.3	cytochrome p450
CUST_21988_PI427639955	Aalb_oocyte_rep_c8445 (GSTD1)			2.1	glutathione s-transferase d1
CUST_7021_PI427639955	Aalb_oocyte_GH79BIP02JQ8K1 (CYP6N17 in *Ae. aegypti*)			2.0	cytochrome p450

Table S5: Top 20 commonly down-regulated probes (C-S) in all three locations in comparison with susceptible VCRU strain FC>2 ($p < 0.01$).

Probe name	Gene-ID	Absolute – FC			Description
		Penang	Johor Bharu	Kuala Lumpur	
CUST_22873_PI427639955	Aalb_oocyte_c33725	143.4	26.4	47.9	vitellogenin-a1
CUST_7438_PI427639955	Aalb_oocyte_rep_c39281	59.0	55.7	19.5	vitelline membrane protein homolog
CUST_18649_PI427639955	Aalb_oocyte_rep_c61804	57.1	59.6	13.2	vitelline membrane protein homolog
CUST_22229_PI427639955	Aalb_oocyte_rep_c13033	47.4	31.7	20.7	cathepsin b
CUST_20753_PI427639955	Aalb_oocyte_rep_c16648	45.6	53.7	14.9	---NA---
CUST_6501_PI427639955	Aalb_oocyte_rep_c46407	36.4	35.7	8.1	vitelline membrane protein homolog
CUST_21152_PI427639955	Aalb_oocyte_rep_c7314	30.8	29.0	12.2	vitellogenin-a1
CUST_48_PI427639955	Aalb_oocyte_rep_c14495	29.5	74.0	6.4	serine threonine-protein kinase rio2
CUST_23818_PI427639947	AAEL010434-RA	25.8	20.0	10.6	Vitellogenin-A1 Precursor (VG)(PVG1)
CUST_28769_PI427639947	AAEL017403-RA	22.9	24.1	7.5	Vitelline membrane protein 15a-2 Precursor
CUST_28768_PI427639947	AAEL017403-RA	22.8	22.8	7.5	Vitelline membrane protein 15a-2 Precursor
CUST_22248_PI427639947	AAEL006670-RA	19.1	21.3	5.5	conserved hypothetical protein
CUST_7439_PI427639955	Aalb_oocyte_rep_c39281	16.3	18.5	13.1	vitelline membrane protein homolog
CUST_16494_PI427639955	Aalb_oocyte_rep_c47052	11.6	11.5	6.2	vitelline membrane protein homolog

Table S6: Mortality of *CYP6P12* transgenic and control *Drosophila* flies after exposure to each of the 5 insecticides

	**Average % Mortality at Each Measured Duration of Exposure**					
**Exposed Group**	**1 Hour**	**2 Hour**	**3 Hour**	**6 Hour**	**24 Hour**	**48 Hour**
**2% Permethrin**						
GAL4-Actin/UAS-NO ♀	4.5^{ns}	26.7^{ns}	43.5^{ns}	47.1^{ns}	68.4^{ns}	NR
GAL4-Actin/UAS-6P12 ♀	4.8^{ns}	29.9^{ns}	41^{ns}	53.5^{ns}	79.7^{ns}	NR
GAL4-Actin/UAS-NO ♂	66.1^{ns}	93.2^{ns}	95.5^{ns}	95.5^{ns}	97.7^{ns}	NR
GAL4-Actin/UAS-6P12 ♂	59.3^{ns}	96.3^{ns}	100^{ns}	100^{ns}	100^{ns}	NR
**0.15% Deltamethrin**						
GAL4-Actin/UAS-NO ♀	35.5	77	84	87.5	100^{ns}	NR
GAL4-Actin/UAS-6P12 ♀	2.5***	2.5***	5.8***	16.8***	98.3^{ns}	NR
**0.005% Bendiocarb**						
GAL4-Actin/UAS-NO ♀	0	0	0	0^{ns}	27.4^{ns}	NR
GAL4-Actin/UAS-6P12 ♀	0	0	0	2.2^{ns}	41.9^{ns}	NR
**0.01% Bendiocarb**						
GAL4-Actin/UAS-NO ♀	5.4^{ns}	32.1^{ns}	49.6^{ns}	98.3^{ns}	98.3^{ns}	NR
GAL4-Actin/UAS-6P12 ♀	24.6^{ns}	39.1^{ns}	48.1^{ns}	64.4^{ns}	88.2^{ns}	NR
GAL4-Actin/UAS-NO ♂	53.4^{ns}	100	100^{ns}	100	100	NR
GAL4-Actin/UAS-6P12 ♂	13.5^{ns}	41.6*	81.6^{ns}	100	100	NR
**2% Etofenprox**						

GAL4-Actin/UAS-NO ♂	12.9^{ns}	15.7^{ns}	21.3	21.3	NR	NR
GAL4-Actin/UAS-6P12 ♂	0^{ns}	0^{ns}	0*	0*	NR	NR
**0.2% Bifenthrin**						
GAL4-Actin/UAS-NO ♀	0^{ns}	0^{ns}	0^{ns}	10.8^{ns}	NR	63.9**
GAL4-Actin/UAS-6P12 ♀	1.7^{ns}	1.7^{ns}	3.3^{ns}	18.2^{ns}	NR	89.2
GAL4-Actin/UAS-NO ♂	0^{ns}	27.1^{ns}	31.3^{ns}	39.6^{ns}	NR	92.5^{ns}
GAL4-Actin/UAS-6P12 ♂	5.3^{ns}	57.8^{ns}	61.7^{ns}	77^{ns}	NR	97.3^{ns}

ns; not significant

Table S7: Summary statistics for polymorphism of *CYP6P12* and *CYP6N3* between the four *Ae. albopictus* populations from Malaysia

Samples	N	S	h	Syn	Nonsyn	π (k)	D (Tajima)	D* (Fu and Li)
					**CYP6P12**			
**KL**	5	15	2	13	2	0.0059(9)	1.83^{ns}	1.83*
**JB**	5	85	5	74	12	0.028 (42)	0.26^{ns}	0.28^{ns}
**KB**	5	1	2	0	1	0.00026(0.4)	-0.82^{ns}	-0.82^{ns}
**PG**	4	0	1	0	0	0	nd	nd
**Total**	19	129	9	114	119	0.027(42)	0.44^{ns}	0.45^{ns}
					**CYP6N3**			
**KL**	5	34	4	26	6	0.0089 (13.4)	-095^{ns}	-095^{ns}
**JB**	4	37	4	29	8	0.013(19.8)	-0.18^{ns}	-0.18^{ns}
**KB**	5	42	3	34	8	0.015(22)	0.98^{ns}	0.98^{ns}
**PG**	4	69	4	50	20	0.025(38.2)	-0.004^{ns}	0.04^{ns}
**Total**	18	88	13	66	25	0.017 (25)	-0.21^{ns}	-0.62^{ns}

N= number of sequences (n); S, number of polymorphic sites; Syn, Synonymous mutations; Nonsyn, Non-synonymous mutations; π, nucleotide diversity (k= mean number of nucleotide differences); Tajima's D and Fu and Li's D statistics, ns, not significant.

Table S8: Binding parameters of the productive mode of various insecticides docked to the active sites of *CYP6P12*

Ligand	Atoms	Weight	Score	Flexible bonds	Hydrogen bond score	Steric interaction score	Ligand conformation penalty	RMSD (Å)
Permethrin	46	391.29	-86.04	7	0.00	-91.19	5.15	32.11
Deltamethrin	47	505.21	-79.03	8	0.00	-86.97	7.94	32.93
Bifenthrin	51	422.87	-81.01	7	-2.45	-85.82	7.26	29.82
λ-cyhalothrin	50	449.85	-83.75	9	0.00	-92.98	9.23	32.01
Bendiocarb	29	223.23	-56.49	2	-1.89	-56.86	2.26	31.70
DDT	28	354.48	-68.60	3	0.00	-69.56	0.96	32.04

Table S9: Primers used for qRT-PCR for microarray candidate genes validation.

Primer Name	Sequence	Product size (base pairs)	Efficiency (%)
qAlbCYP6N3 F	AAAATTGCATAAATGAAACTCTTCGTAAA	124	94.9
qAlbCYP6N3 R	ATCATCACCGACGTGCCTTT		
qAlbCYP9AE1 F	TTGGAATGACGACGAGTTGA	132	110.2
qAlbCYP9AE1 R	TCGAATAACCGCTCCTGAAT		
qAlbGSTT3 F	GAGGAAATTTGAAAACCGTTCGTC	133	92.2
qAlbGSTT3 R	GAACTCCCGCGACAGATACC		
qAlbCYP9J17 F	TGGATCGTTTGTGATCGAGA	148	103.6
qAlbCYP9J17 R	AGGCGTACGGATTGATCTTC		
qAlbABCA F	CTTTGGATTGTTAGGAATGAACGGA	150	89.3
qAlbABCA R	TAACCGTACTGGGAGCGGTA		
qAlbCYP6M2 F	TCACACTGGAGAAGGACTGC	117	90.0
qAlbCYP6M2 R	CACTCTGGTCCGGATTGAAG		
qAlbGSTD1 F	GGGTCCAGTTGAACCTGAAG	144	98.6
qAlbGSTD1 R	TTTGAATGGCTCTGCTTTCC		
qAlbCYP6P12 F	CGTGCGCTTTTGGGATTGAG	145	94.7
qAlbCYP6P12 R	ATCGTCCGTGCCAAATCCTT		
qAlbSCD01845 F	GACCGCCAAGAATGGGAAGA	134	95.4
qAlbSCD01845 R	AGCAAACAAGCCTTCGAGGT		
qAlbCYP9M6 F	AGTTGGCAGCTACTGGAGGA	116	105.2
qAlbCYP9M6 R	GAAATCAGCTGCTTCCTTGG		
qAlbSCD15871 F	GAGGAACGTTCCTAGTATCCAAGG	110	105.8
qAlbSCD15871 R	GGCGAACCATTCCGGTTCTA		
qTub-Aae F	CCGCACTCGAGAAGGATTAC	131	102.0
qTub-Aae R	GTGGTTCGGTTTGACTTCGT		
qRPS7-Aae F	AAGGTCGACACCTTCACGTC	131	95.1
qRPS7-Aae R	CGCGCGCTCACTTATTAGAT		

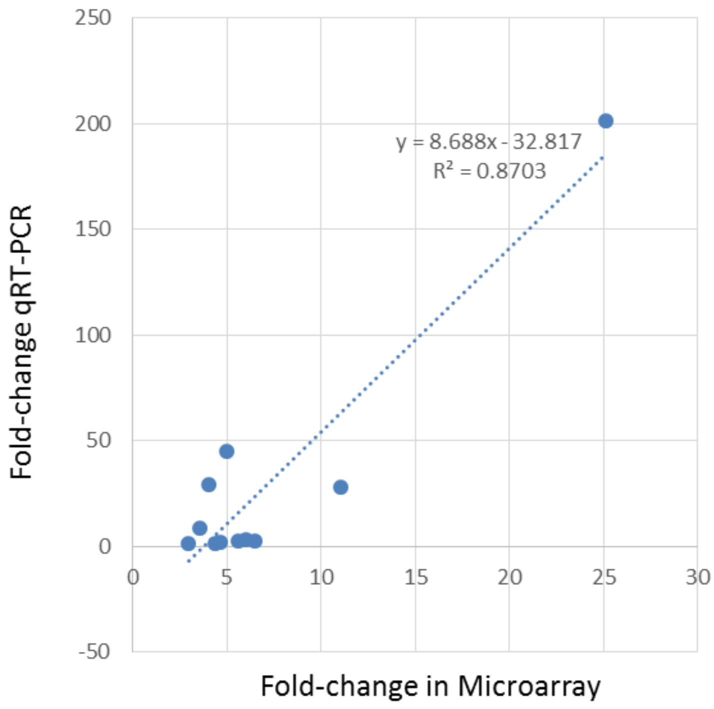

Figure S1: Transcriptomic analysis: Correlation between microarray data and qRT-PCR results.

```
        10        20        30        40        50        60        70        80
....|....|....|....|....|....|....|....|....|....|....|....|....|....|....|....|
  MLAYLLAVVVALLTAAALYIRSRHRFWSDRGIPCAPNPEFLLGHVRGVVTNKHAAHVNRELYQDFKKRGESFGGFSFF
        90       100       110       120       130       140       150       160
....|....|....|....|....|....|....|....|....|....|....|....|....|....|....|....|
AVPAVLVVDPELVKTMLVRDFAVFHDRGIYNNPKDDPLSGQLFLLEGLQWRVLRQMLTPTFTSGRMKAMFGTIMDVAEEF
       170       180       190       200       210       220       230       240
....|....|....|....|....|....|....|....|....|....|....|....|....|....|....|....|
RRFMVECRERESVIEMKEVLASFTTDVIGTCAFGIECNTLKNPDSDFLKYGKKVFEQKLSTLVKFIFASLFKDLARTMGI
       250       260       270       280       290       300       310       320
....|....|....|....|....|....|....|....|....|....|....|....|....|....|....|....|
KITDAGVEKFFMGLVRETVEFREKHNVQRNDFMNLLLQLKNKGRLVDQLDEAEEAAARGLTMDELAAQCFVFFIAGYETS
       330       340       350       360       370       380       390       400
....|....|....|....|....|....|....|....|....|....|....|....|....|....|....|....|
STTMNFCLYELAKNPDIQERLREDIEEAVAVNDGQVTYDLVMGLRYLDNVVNETLRKYPPIESLNRVPNSDYTVPGTNHV
       410       420       430       440       450       460       470       480
....|....|....|....|....|....|....|....|....|....|....|....|....|....|....|....|
LPAQTMITIPVYALHHDPDYYPDPDRFDPDRFLPEVVQGRHPYAYIPFGEGPRNCIGMRFGVMQTKIGLLTLLRNFRFSP
       490       500       510
....|....|....|....|....|....|
SPRTPDKIVFDVKSFVLSPEGGNYLRYDKI
```

Figure S2: Full-length amino acid sequence of the CYP6P12 allele used to generate the transgenic *Drosophila* strain.

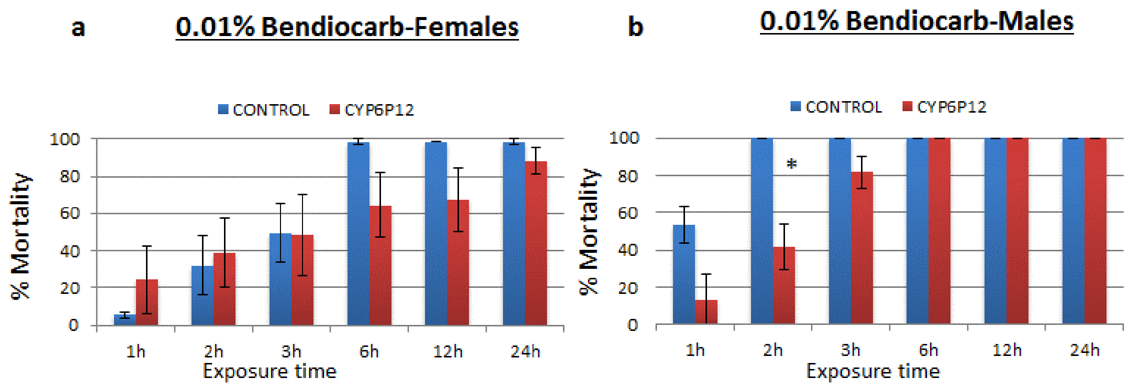

Figure S3: Knockdown and mortality rate after bioassay tests with transgenic strains for CYP6P12 with bendiocarb. A) is the result for test with 0.01% bendiocarb on the females of the transgenic Act5C-CYP6P12 strain and the control strain (the progeny from the cross between the UAS-CYP6P12 females and w^{1118} males (which do not over-express the P450 transgene). B) is for males.
